# Validation of a non‐conforming monolithic fluid‐structure interaction method using phase‐contrast MRI

**DOI:** 10.1002/cnm.2845

**Published:** 2017-02-16

**Authors:** Andreas Hessenthaler, Oliver Röhrle, David Nordsletten

**Affiliations:** ^1^ Institute of Applied Mechanics (CE) University of Stuttgart Pfaffenwaldring 7 70569 Stuttgart Germany; ^2^ Division of Imaging Sciences and Biomedical Engineering King's College London 4th Floor, Lambeth Wing St. Thomas Hospital London, SE1 7EH UK

**Keywords:** ALE, monolithic FSI, non‐conforming, validation, 3D FSI experiment

## Abstract

This paper details the validation of a non‐conforming arbitrary Lagrangian‐Eulerian fluid‐structure interaction technique using a recently developed experimental 3D fluid‐structure interaction benchmark problem. Numerical experiments for steady and transient test cases of the benchmark were conducted employing an inf‐sup stable and a general Galerkin scheme. The performance of both schemes is assessed. Spatial refinement with three mesh refinement levels and fluid domain truncation with two fluid domain lengths are studied as well as employing a sequence of increasing time step sizes for steady‐state cases. How quickly an approximate steady‐state or periodic steady‐state is reached is investigated and quantified based on error norm computations. Comparison of numerical results with experimental phase‐contrast magnetic resonance imaging data shows very good overall agreement including governing of flow patterns observed in the experiment.

## 1 INTRODUCTION

Many industrial and engineering problems, for example, in aeronautics,^1^ power generation,^2^ defense,^3^ and biomedical engineering,^4^, ^5^ involve complex multiphysics phenomena, such as the interaction between fluids and solids. In the field of biomedical engineering, collaborative work of researchers, modelers, physicians, medical imaging technicians, and others becomes increasingly important to ultimately provide patient‐specific models^6^, ^7^, ^8^, ^9^ for therapy planning.

Innumerable mathematical modeling techniques and numerical solution methods for fluid‐structure interaction (FSI) problems have been proposed to date and can be classified based on the nature of the underlying algorithmic approach and solution strategy. Classification based on the nature of underlying meshes distinguishes immersed methods and moving domain methods. Immersed methods employ a fixed background mesh and a Eulerian description for the motion of the fluid over the flow domain, whereas the solid motion and deformation are described within a Lagrangian coordinate frame on an embedded mesh. The presence of the solid is accounted for either via adding a body force term to the fluid equations (immersed boundary method^10^) to constrain local flow with a similar effect as the no‐slip condition at fluid‐solid boundaries, or by coupling the fluid and solid equations by introducing a Lagrange multiplier at the fluid‐solid interface (ficticious domain method^11^, ^12^). Immersed methods are particularly well suited for FSI problems involving large structural deformation or solids moving through flow domains. On the other hand, moving domain methods enable inherent interface‐tracking by using an arbitrary Lagrangian‐Eulerian (ALE) coordinate frame for the fluid domain^13^ and constraining the fluid and solid equations by requiring equal but opposite tractions and the no‐slip condition at the fluid‐solid interface. Although, the ability of this method to deal with large structural deformation is often noted as a limitation, and remeshing steps might become necessary to maintain mesh quality. On the other hand, Lagrangian meshless methods, such as the study of Idelsohn et al,^14^ avoid the requirement for remeshing. Fluid‐structure interaction methods can also be classified based on the solution strategy, for example, monolithic/partitioned approach (global assembly and solution of a single matrix system *vs* solution of fluid and solid subsystems with exchange of boundary values) and explicit/implicit discretization and time integration, where a given choice may impact computational cost, accuracy, and stability.

In this paper, we consider a monolithic ALE FSI technique that is able to use non‐conforming meshes at the interface,^5^, ^15^, ^16^, ^17^ such that meshes can be designed based on the requirements of the physics of the coupled subsystems leading to improved accuracy and to decreased computational cost (by avoiding underrefinement and overrefinement, respectively). Coupling of subdomain equations is achieved via introduction of an additional coupling domain and enforcing interface constraints by means of a Lagrange multiplier variable. The method has been studied regarding stability and convergence^15^, ^18^ and successfully applied to various biomedical engineering problems, such as the simulation of whole‐heart and left ventricular mechanics.^15^, ^16^, ^17^, ^19^, ^20^ Previously, the method has been used for coupling the non‐conservative ALE Navier‐Stokes equations and the governing equations for quasi‐static/transient finite elasticity. It has been extended recently to enable modeling of turbulent flow phenomena by a stabilized cG(1)cG(1) scheme^21^ to extend the use of the method over a larger range of Reynolds numbers. Besides the various biomedical engineering applications,^5^, ^17^, ^20^ the method has been assessed and verified using test problems; however, it was not validated in any previous work. Thus, validation of the method will be the focus of this work as well as validation of using the cG(1)cG(1) scheme within the Lagrange multiplier‐based coupling method. In this work, we focus on the validation of the method as well as the comparative performance of an inf‐sup stable scheme and the cG(1)cG(1) approach.

Verification and validation are important to confirm fidelity and assess the capabilities of established and newly proposed mathematical models and numerical algorithms. Thus, standard numerical benchmark problems^22^, ^23^, ^24^, ^25^, ^26^ and FSI experiments^27^, ^28^, ^29^, ^30^, ^31^, ^32^, ^33^, ^34^, ^35^ have been developed over the last decades and found widespread use. In this tradition, a recently developed 3D FSI experiment^36^, ^37^ introduces two new challenging benchmark test cases that involve steady and periodic interaction between a moderately viscous incompressible fluid and an incompressible nonlinear solid in a 3D setting.^36^, ^37^ With key aspects (for example, flow regime, material parameters, and mechanical properties) of the experiment being in line with those given in typical translational biomedical engineering applications (such as simulation of left ventricular mechanics under support of a left ventricle assist device^5^), the benchmark is considered in this paper for validation of the non‐conforming monolithic FSI method. An inf‐sup stable (iss) and the cG(1)cG(1) scheme are considered for the fluid model. Numerical predictions of both methods are compared and contrasted with experimental phase‐contrast magnetic resonance imaging (MRI) data. Spatial refinement, fluid domain truncation, and convergence to (periodic) steady‐state are studied in this paper. Further, the employed coupling technique is assessed, and performance of both schemes is investigated regarding computational cost and prediction of steady and dynamic behavior (flow patterns, deformation of solid, summed forces at fluid / solid boundary, and others).

In the following, the numerical solution procedure is based on the use of a non‐conforming monolithic ALE FSI technique. The details of this method are outlined in Section 2. Further, in Section 3, we present results obtained for both benchmark test cases and validate our results via comparison with experimental results. Further, aspects such as spatial refinement and early truncation of the fluid domain are assessed. Finally, we discuss the quality of our numerical results and conclude with possible future improvements in Section 4.

## 2 METHODOLOGY

In this section, a brief overview of the 3D FSI experiment is given. The model problem and the respective model equations are detailed. The weak form for the FSI problem within a finite element formulation using Lagrange multipliers is given using an inf‐sup stable and stabilized scheme. The section concludes with details about the numerical solution and the definition of error norms for comparison of numerical and experimental data.

### 2.1 3D FSI experiment

**Figure 1:**
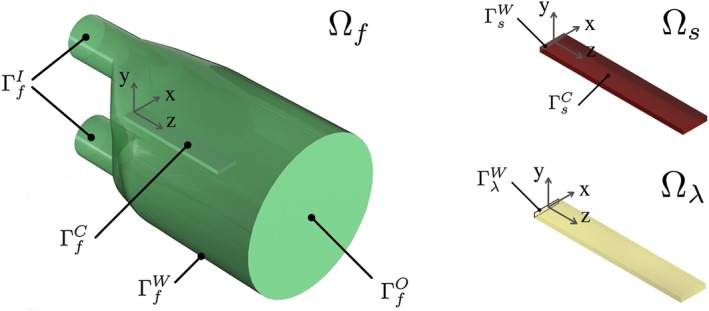
3D FSI benchmark subdomains: flow domain Ω_*f*_, solid domain Ω_*s*_, and coupling domain Ω_*λ*_

The flow domain in the relevant section (that is, where FSI phenomena occur) of the experiment features two inlets (diameter ⊘21.9 mm) that merge smoothly into a single outlet (diameter ⊘76.2 mm), with a solid (volume 11 × 2 × 65 mm^3^) attached to the wall in the merging section (Figure 1). A right‐handed Cartesian coordinate system is used^37^ with the origin chosen to be at the center of the attachment point of the solid to the wall.

The selected pump rates create steady (Phase I) and periodic (Phase II) inflow. This yields steady and time‐dependent periodic interaction between a moderately viscous incompressible fluid (aqueous glycerol solution) and an incompressible nonlinear solid (silicone material).

Experimental data were acquired using MRI techniques and are available for comparison with numerical results. The data include the geometry under zero inflow conditions (for example, the deformed state of the structure with maximum deflection of 29.50 mm and 25.65 mm for Phases I and II, respectively), inflow boundary condition data and time‐resolved flow and deformation fields.

**Figure 2:**
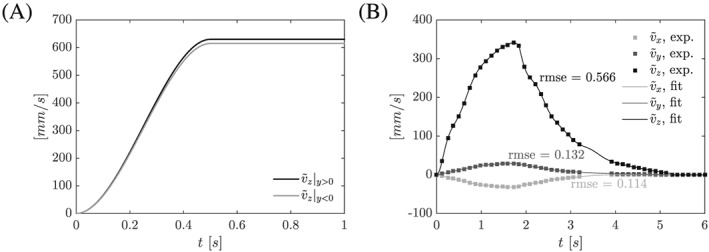
A, Phase I: prescribed peak velocity ${\tilde{v}}_{z}$ for parabolic profile with ${\tilde{v}}_{z}{|}_{y>0}=630$ mm/s and ${\tilde{v}}_{z}{|}_{y<0}=615$ mm/s for t⩾0.5 s (Equation (1)) and ${\tilde{v}}_{x}={\tilde{v}}_{y}=0\;\forall \;t\in [0,T]$. B, Phase II: recorded peak velocity ${\tilde{v}}_{i}$ (*i*∈{*x*,*y*,*z*}) for parabolic inflow and data fit. We note, that ${\tilde{v}}_{y}{|}_{y<0}=0$ 37

For Phase I, parabolic inflow profiles ${\tilde{v}}_{f}^{I}$ were defined on $\Gamma_{f}^{I}$ with peak value ${\left[0,0,{\tilde{v}}_{z}\right]}^{T}$

(1) $${\tilde{v}}_{z}=\left\{\begin{array}{cc} 630\cdot (24t^{3}-8t^{2})\; & y>0,t<0.5,\\ 615\cdot (24t^{3}-8t^{2}) & y<0,t<0.5,\\ 630 & y>0,t⩾0.5,\\ 615 & y<0,t⩾0.5, \end{array}\right.$$

with a smooth increase over 0.5 s (Figure 2A) for the upper (*y* > 0) and lower (*y* < 0) inlet.

For Phase II, parabolic profiles ${\tilde{v}}_{f}^{I}$ were defined on $\Gamma_{f}^{I}$ with peak values ${\left[{\tilde{v}}_{x},{\tilde{v}}_{y},{\tilde{v}}_{z}\right]}^{T}$ fit to the experimental data, see Figure 2B.

Fluid material parameters and solid density are defined in the study of Hessenthaler et al^37^ and collected in Table 1, which also contains the solid material parameter for an isotropic incompressible Neo‐Hookean material law. It was shown in the study of Hessenthaler et al^37^ that this solid constitutive model can be used to model the mechanical response of the silicone material and that it is able to represent test data obtained from a uniaxial tensile load‐displacement test. However, the silicone material used in the FSI experiment undergoes a continuous curing process,^37^ such that the solid material model was calibrated using the zero inflow displacement data recorded for both test cases.

**Table 1:** Material parameters

**Material parameter**	**Phase I**	**Phase II**	**Unit**
Fluid density *ρ*_*f*_	1163.3	1164.0	[kg/m^3^]
Dynamic viscosity *μ*_*f*_	12.50	13.37	[mPa· s]
Kinematic viscosity *ν*_*f*_	10.75	11.48	[mm^2^/s]
Solid density *ρ*_*s*_	1058.3	1058.3	[kg/m^3^]
Neo‐Hookean parameter *μ*_*s*_	61	74	[kPa]

Fluid material parameters and solid density were taken from the studies of Gaddum et al and Hessenthaler et al.^36^, ^37^ Neo‐Hookean parameter was selected to reproduce zero inflow displacement.

### 2.2 Reference frames

The 3D FSI domain arising from the definition of the 3D FSI experiment consists of the fluid and solid subdomains, $\Omega_{f}\subset R^{3}\times Ī$ and $\Omega_{s}\subset R^{3}\times Ī$ (with Ī=[0,T]), and its respective subdomain boundaries, Γ_*f*_ and Γ_*s*_. The fluid domain boundary $\Gamma_{f}=\Gamma_{f}^{I}\cup \Gamma_{f}^{O}\cup \Gamma_{f}^{W}\cup \Gamma_{f}^{C}$ is partitioned into inlet $\Gamma_{f}^{I}$, outlet $\Gamma_{f}^{O}$, wall $\Gamma_{f}^{W}$, and coupling interface $\Gamma_{f}^{C}$ subdomains, see Figure 1. Similarly, the solid domain boundary $\Gamma_{s}=\Gamma_{s}^{W}\cup \Gamma_{s}^{C}$ is partitioned into subdomains for the wall $\Gamma_{s}^{W}$ and the coupling surface $\Gamma_{s}^{C}$. A third coordinate frame on the coupling domain $\Omega_{\lambda }=\Gamma_{f}^{C}=\Gamma_{s}^{C}$ is introduced to enforce coupling of the fluid and solid equations. Further, $\Omega_{i}^{0}$ denotes reference domain *i* (with respective boundary $\Gamma_{i}^{0}$), and ***n***_*i*_ denotes the outward boundary normal.

To relate reference domains, $\Omega_{i}^{0}$, to moving domains, Ω_*i*_(*t*), bijective mappings are introduced,^38^, ^39^

(2) $$A_{f}(X,t)=u_{f}(X,0)+\int_{0}^{t}w_{f}(X,t)\;dt+X,$$

(3) $$L_{s}(X,t)=u_{s}(X,t)+X,$$

where ***w***_*f*_ is the fluid domain velocity (referred to as ALE velocity), ***u***_*i*_ is the deformation of the domain (generally, ***u***_*i*_(·,0) ≠ 0 such that $\Omega_{i}^{0}\neq \Omega_{i}(0)$ and $\Gamma_{i}^{0}\neq \Gamma_{i}(0)$), and ***X*** is the spatial coordinate of a point in the reference domain. More precisely, Equations (2) and (3) denote an ALE mapping for the fluid domain and a Lagrangian mapping for the solid domain, repectively, such that conservation laws can be equivalently formulated on the reference or moving domain. Using the ALE and Lagrangian mapping, we can link a function given on reference domain $\Omega_{f}^{0}$ to its counterpart on the moving domain Ω_*f*_, ie,

(4) $$\hat{f}(x,t)=f(X,t),\;x=A(X,t)\;\text{in}\;\Omega_{f}^{0}\times [0,T],$$

where ***x*** is the spatial coordinate of a point in the moving domain and likewise for functions given on $\Omega_{s}^{0}$ and Ω_*s*_. Further, the Jacobian mapping of the domain displacement is given as $J_{i}:=\det F_{i}$, where ***F***_*i*_ = ∇_***X***_(***u***_*i*_ + ***X***) is the deformation gradient tensor. In the following, the *hat* notation in Equation (4) will be omitted; *∂*_*t*_ denotes the temporal derivative with respect to a fixed point in the reference domain (eg, the studies of Nordsletten et al,^16^, ^19^ and Formaggia and Nobile^40^), and ∇_***x***_ and ∇_***X***_ are the Eulerian and Lagrangian gradient operators.

### 2.3 FSI model problem

The non‐conservative ALE Navier‐Stokes equations 19, 38, 40, 41, 42 and the governing equations for finite elasticity^43^, ^44^ are employed to model incompressible Newtonian fluid flow on the moving domain Ω_*f*_ and the deformation of an incompressible nonlinear solid material on Ω_*s*_. Dynamic and kinematic interface constraints are used to couple the fluid and solid equations. An anisotropic diffusion model is selected to model the fluid domain deformation by determining the ALE velocity ***w***_*f*_ in Equation (2). Then, the fluid velocity and pressure variables, ***v***_*f*_ and *p*_*f*_; the solid velocity, displacement, and pressure variables, ***v***_*s*_, ***u***_*s*_, and *p*_*s*_; and the ALE velocity ***w***_*f*_ satisfy

(5) $$\begin{array}{cc} \rho_{f}\;\partial_{t}v_{f}+\rho_{f}(v_{f}-w_{f})\cdot \nabla_{x}v_{f}-\nabla_{x}\cdot \sigma_{f} & =0\;\text{in}\;\Omega_{f},\\ \nabla_{x}\cdot v_{f} & =0\;\text{in}\;\Omega_{f},\\ v_{f} & =v_{f}^{d}\;\text{on}\;\Gamma_{f}^{I}\cup \Gamma_{f}^{W},\\ \sigma_{f}\cdot n_{f} & =t_{f}^{n}\;\text{on}\;\Gamma_{f}^{O},\\ v_{f}(\cdot ,0) & =0\;\text{on}\;\Omega_{f}(0), \end{array}$$

(6) $$\begin{array}{cc} \rho_{s}\;\partial_{t}v_{s}-\nabla_{x}\cdot \sigma_{s} & =(\rho_{s}-\rho_{f})\;g\;\text{in}\;\Omega_{s},\\ J_{s}(u_{s})-1 & =0\;\text{in}\;\Omega_{s}^{0}\times [0,T],\\ u_{s}=v_{s} & =0\;\text{on}\;\Gamma_{s}^{W},\\ v_{s}(\cdot ,0) & =0\;\text{on}\;\Omega_{s}^{0},\\ u_{s}(\cdot ,0) & =u_{0}\;\text{on}\;\Omega_{s}^{0}, \end{array}$$

(7) $$\begin{array}{cc} \partial_{t}w_{f}+\nabla_{X}\cdot (\Phi \nabla_{X}w_{f}) & =0\;\text{in}\;\Omega_{f}^{0}\times [0,T],\\ w_{f} & =w_{f}^{d}\;\text{on}\;\Gamma_{f},\\ w_{f}(\cdot ,0) & =0\;\text{on}\;\Gamma_{f}^{0}, \end{array}$$

(8) $$\begin{array}{cc} \sigma_{f}\cdot n_{f}+\sigma_{s}\cdot n_{s} & =0\;\text{on}\;\Omega_{\lambda },\\ v_{f}-v_{s} & =0\;\text{on}\;\Omega_{\lambda }, \end{array}$$

where *ρ*_*f*_ and *ρ*_*s*_ are the fluid and solid density (Table 1), ***σ***_*f*_ and ***σ***_*s*_ are the Cauchy stress tensors of the fluid and solid, **Φ** = **Φ**(***X***) is a diffusion coefficient tensor, and $\left(v_{f}^{d},w_{f}^{d}\right)$ and $t_{f}^{n}$ are Dirichlet and Neumann boundary condition (BC) data. In our case, $v_{f}^{d}$ and $w_{f}^{d}$ are

$$v_{f}^{d}:=\left\{\begin{array}{ll} {\tilde{v}}_{f}^{I} & \text{on}\;\Gamma_{f}^{I},\\ 0 & \text{on}\;\Gamma_{f}^{W}, \end{array}\right.\;w_{f}^{d}:=\left\{\begin{array}{ll} v_{f} & \text{on}\;\Gamma_{f}^{C},\\ 0 & \text{on}\;\Gamma_{f}^{I}\cup \Gamma_{f}^{O}\cup \Gamma_{f}^{W}, \end{array}\right.$$

with given inflow ${\tilde{v}}_{f}^{I}$(Section 2.1). To be able to deal with potential reflow on the outflow boundary $\Gamma_{f}^{O}$ and prevent backflow divergence, we further introduce outflow stabilization,^45^

(9) $$t_{f}^{n}:=\rho_{f}\;\beta /2\;\left(v_{f}\cdot n_{f}-\left|v_{f}\cdot n_{f}\right|\right)v_{f}\;\text{on}\;\Gamma_{f}^{O},$$

with parameter *β* = 0.2 as suggested in the study of Moghadam et al.^46^

The Cauchy stresses in Equations (5) and (6) are written as

(10) $$\sigma_{f}\;:=\mu_{f}\nabla_{x}v_{f}-\varphi_{f}\;I,\sigma_{s}:=\frac{\mu_{s}}{J^{5/3}}\left(F_{s}F_{s}^{T}-\frac{F_{s}:F_{s}}{3}I\right)-\varphi_{s}I,$$

where *μ*_*f*_ is the dynamic viscosity of the fluid and the silicone material was modeled as an isotropic incompressible Neo‐Hookean material with material parameter *μ*_*s*_(Table 1). Further, a change‐of‐variables (COV) for the pressure variables, *p*_*f*_ and *p*_*s*_,

$$\varphi_{f}=p_{f}-\rho_{f}\;x\cdot g-P_{o},\;\varphi_{s}=p_{s}-\rho_{f}\;x\cdot g-P_{o},$$

was introduced, where *φ*_*f*_ and *φ*_*s*_ are the substituted fluid and solid pressure variables and *P*_*o*_ is the unknown mean outlet pressure. The COV is introduced to account for the contribution of a gravitational field (with ***g*** = [0, − 9.80665,0]^*T*^ m/s^2^) to the momentum balance in Equation (5) (and likewise for Equation (6) to ensure compatibility of stresses). Without COV and under zero inflow conditions, a linear pressure gradient along the *y*‐axis would yield flow in the outlet region due to violation of a zero traction assumption on $\Gamma_{f}^{O}$. On the other hand, the same conditions do not cause flow if a COV is employed.

The *real* pressure is recovered by reversing the COV with

$$p_{f}=\varphi_{f}+\rho_{f}\;x\cdot g+P_{o},p_{s}=\varphi_{s}+\rho_{f}\;x\cdot g+P_{o}.$$

In general, the shape of the fluid domain changes because of the deforming solid. To extend the deformation of the fluid‐solid boundary (with fixed $\Gamma_{f}^{I}\cup \Gamma_{f}^{O}\cup \Gamma_{f}^{W}$) to the interior of the fluid domain, a mesh velocity ***w***_*f*_ is introduced that satisfies Equation (7). Here, the boundary motion is extended to the interior using the typical harmonic extension augmented with a coefficient tensor, **Φ**, to preferentially weight motion in the boundary normal direction. Specifically, the tensor **Φ** was defined as **Φ** = ∇_***X***_ *ψ* ^*T*^∇_***X***_ *ψ* + ***I*** with *ψ* satisfying the Laplacian problem on $\Omega_{f}^{0}$(with *ψ* = 0 on $\Gamma_{f}^{I}\cup \Gamma_{f}^{O}\cup \Gamma_{f}^{W}$ and *ψ* = 80 on Ω_*λ*_). As the variable *ψ* yields a decaying diffusion field from the coupling interface, this introduces anisotropy in **Φ** preferentially distributing motion faster in the direction of ∇_***X***_ *ψ*. The value set for *ψ* was selected based on simple test problems in three dimensions, illustrating preservation of the mesh quality over time.

### 2.4 Finite element formulation using Lagrange multipliers

To couple the fluid and solid models, dynamic and kinematic interface constraints are enforced with equal but opposite tractions, ***t***_*f*_:= ***σ***_*f*_·***n***_*f*_ and ***t***_*s*_:= ***σ***_*s*_·***n***_*s*_, and the no‐slip condition, as detailed in Equation (8). We note that the interface constraints are not influenced by the introduced COV because

$$n_{f}+n_{s}=0\;\text{on}\;\Omega_{\lambda },$$

yielding an equivalent formulation. On the coupling domain Ω_*λ*_, a Lagrange multiplier variable is defined as ***λ*** = ***t***_*f*_ =− ***t***_*s*_, maintaining the interface conditions. Within the continuous weak form for both fluid and solid mechanical subsystems, the resulting boundary terms are substituted with ***λ***, constraining the multiplier through the no‐slip interface condition. Finally, the respective fluid and solid models (given in Equations (5) and (6)) and the interface constraints (Equation (8)) are coupled monolithically. Here, we consider an inf‐sup stable scheme and the cG(1)cG(1) scheme^21^ as a variant of a General Galerkin method. We note, that Equation (7) is partitioned from the main FSI problem and solved in turn.

#### 2.4.1 Spatiotemporal discretization

A first‐order backward Euler scheme is employed for the time discretization, where 0 = *t*_0_ < *t*_1_ < … < *t*_*N*_ = *T* denotes a sequence of discrete time steps with time step size $\Delta_{t}^{n}:=t_{n+1}-t_{n}$.

The fluid domain Ω_*f*_ was discretized using tetrahedral elements, whereas hexahedral elements were selected to discretize the solid domain Ω_*s*_. The coupling domain Ω_*λ*_ was discretized using triangular elements that conform with the fluid domain surface elements on $\Gamma_{f}^{C}$. In the following, we use $\Omega_{i,h}^{0}={\left\{\Omega_{i,h}^{e}\right\}}_{e=1}^{N_{i}^{e}}$ to denote meshes consisting of $N_{i}^{e}$ many, non‐overlapping elements $\Omega_{i,h}^{e}$ and $\Omega_{f,h}^{n}=A\;\left(\Omega_{f,h}^{0},t_{n}\right)$ and $\Omega_{s,h}^{n}=L_{s}\;\left(\Omega_{s,h}^{0},t_{n}\right)$ to denote current fluid and solid meshes.

#### 2.4.2 Weak formulation I: inf‐sup stable scheme

Finite element discretizations were constructed using inf‐sup stable $P^{2}-P^{1}$ Taylor‐Hood elements for fluid velocity and pressure, $P^{2}$ for fluid domain velocity and inf‐sup stable $Q^{2}-Q^{1}$ Taylor‐Hood elements for solid displacement and pressure. Further, $P^{2}$ elements were employed for the Lagrange multiplier variable that was nested into the trace of the richest space on Ω_*λ*_, which was on the fluid side. The discrete solution at each step *n* in time can then be written as follows:

Find $s^{n+1}:=\left(v_{f}^{n+1},v_{s}^{n+1},\lambda^{n+1},\varphi_{f}^{n+1},\varphi_{s}^{n+1}\right)\in S_{D}^{h}:=V_{D}^{h}\times U_{D}^{h}\times M_{0}^{h}\times W_{f}^{h}\times W_{s}^{h}$ and $w_{f}^{n+1}\in W_{D}^{h}$, such that for every $d:=(y,w,q,q_{f},q_{s})\in S_{0}^{h}:=V_{0}^{h}\times U_{0}^{h}\times M_{0}^{h}\times W_{f}^{h}\times W_{s}^{h}$ and $z\in W_{0}^{h}$:

$$\begin{array}{cc} R\;\left(s^{n+1},s^{n},w_{f}^{n+\theta };d\right):= & \int_{\Omega_{f,h}^{n+1}}\rho_{f}\left[\frac{v_{f}^{n+1}-v_{f}^{n}}{\Delta_{t}^{n}}+\left(v_{f}^{n+\theta }-w_{f}^{n+\theta }\right)\cdot \nabla_{x}v_{f}^{n+\theta }\right]\cdot y\;dx\\ & +\int_{\Omega_{f,h}^{n+1}}\sigma_{f}^{n+\theta }:\nabla_{x}y+q_{f}\nabla_{x}\cdot v^{n+\theta }\;dx\\ & +\int_{\Omega_{s,h}^{0}}J_{s}^{n+1}\left[\rho_{s}\frac{v_{s}^{n+1}-v_{s}^{n}}{\Delta_{t}^{n}}-(\rho_{s}-\rho_{f})g\right]\cdot w\;dX\\ & +\int_{\Omega_{s,h}^{0}}P_{s}^{n+\theta }:\nabla_{X}w+q_{s}\left(J_{s}^{n+\theta }-1\right)\;dX\\ & +\int_{\Omega_{\lambda ,h}^{0}}\lambda^{n+\theta }\cdot (y-w)+q\cdot \left(v_{f}^{n+\theta }-v_{s}^{n+\theta }\right)\;dX=0,\\ R_{w_{f}}\left(w_{f}^{n+1},w_{f}^{n};z\right):= & \int_{\Omega_{f,h}^{0}}\left[\frac{w_{f}^{n+1}-w_{f}^{n}}{\Delta_{t}^{n}}\cdot z-\left(\Phi \nabla_{X}w_{f}^{n+\theta }\right):\nabla_{X}z\right]\;dX=0, \end{array}$$

where $P_{s}^{n+\theta }={\left(\sigma_{s}F_{s}^{-T}/J_{s}\right)}^{n+\theta }$ is the first Piola‐Kirchhoff stress tensor, $u^{n+\theta }=u^{n}+\theta \Delta_{t}^{n}v^{n+1}$, and the notation *Y* ^*n* + *θ*^ = *θ* *Y* ^*n* + 1^ + (1 − *θ*)*Y* ^*n*^.

Following the study of Nordsletten et al,^16^ we introduce,

$$\begin{array}{cc} S^{k}\left(\Omega_{i,h}^{0}\right) & =\left\{y:\;\Omega_{i,h}^{0}\to R\;|\;y\in C\left(\bar{\Omega_{i,h}^{0}}\right),\right.\\ & \;\left.y{|}_{\Omega_{i,h}^{e}}\in P^{k}\left(\Omega_{i,h}^{e}\right),\forall \;\Omega_{i,h}^{e}\subset \Omega_{i,h}^{0}\right\}, \end{array}$$

defining all *k* ^*th*^‐order piecewise continuous polynomials on respective discretized domains (*i* = *f*,*s*), where $P^{k}$ are the polynomials of degree k,k⩾1. Finally, from the spaces,

$$\begin{array}{cc} & V^{\;h}={\left[S^{2}\left(\Omega_{f,h}^{0}\right)\right]}^{3},\;U^{\;h}={\left[S^{2}\left(\Omega_{s,h}^{0}\right)\right]}^{3},\\ & W_{f}^{h}=S^{1}\left(\Omega_{f,h}^{0}\right),\;W_{s}^{h}=S^{1}\left(\Omega_{s,h}^{0}\right), \end{array}$$

we select only those functions satisfying the Dirichlet BC or homogeneous BC,

(11) $$\begin{array}{cc} V_{D}^{\;h} & =\left\{y\in V^{h}\;|\;y=v_{f}^{d}\;\;\text{on}\;\Gamma_{f,h}^{I}\cup \Gamma_{f,h}^{W}\right\},\\ V_{0}^{\;h} & =\left\{y\in V^{h}\;|\;y=0\;\;\text{on}\;\Gamma_{f,h}^{I}\cup \Gamma_{f,h}^{W}\right\}, \end{array}$$

(12) $$\begin{array}{cc} U_{D}^{\;h} & =\left\{y\in U^{\;h}\;|\;y=0\;\;\text{on}\;\Gamma_{s,h}^{W}\right\},\\ U_{0}^{\;h} & =U_{D}^{\;h}, \end{array}$$

(13) $$\begin{array}{cc} W_{D}^{\;h} & =\left\{y\in V^{\;h}\;|\;y=w_{f}^{d}\;\;\text{on}\;\Gamma_{f,h}\right\},\\ W_{0}^{\;h} & =\left\{y\in V^{\;h}\;|\;y=0\;\;\text{on}\;\Gamma_{f,h}\right\}, \end{array}$$

and define the Lagrange multiplier space as,

$$M_{0}^{h}=\left\{z\in \gamma_{\Gamma_{f,h}^{C}}V_{0}^{h}\right\},$$

where $\gamma_{\Gamma_{f,h}^{C}}$ is the trace operator on $\Gamma_{f,h}^{C}$.

#### 2.4.3 Weak formulation II: cG(1)cG(1) scheme

Here, the cG(1)cG(1) scheme as given for the Navier‐Stokes equations in the study of Hoffman et al^21^ is considered to be able to employ equal‐order interpolation and to govern potential turbulent effects. Finite element discretizations were constructed using $P^{1}-P^{1}$ Taylor‐Hood elements for fluid velocity and pressure, $P^{1}$ elements for fluid domain velocity and inf‐sup stable $Q^{2}-Q^{1}$ Taylor‐Hood elements for solid displacement and pressure. Further, $P^{1}$ elements were employed for the Lagrange multiplier variable, that was again nested into the trace of the richest space on Ω_*λ*_, which was on the fluid side. The discrete solution at each step *n* in time can then be written as follows:

Find $s^{n+1}\in {\tilde{S}}_{D}^{h}:={\tilde{V}}_{D}^{h}\times U_{D}^{h}\times {\tilde{M}}_{0}^{h}\times W_{f}^{h}\times W_{s}^{h}$ and $w_{f}^{n+1}\in {\tilde{W}}_{D}^{h}$, such that for every $d\in {\tilde{S}}_{0}^{h}:={\tilde{V}}_{0}^{h}\times U_{0}^{h}\times {\tilde{M}}_{0}^{h}\times W_{f}^{h}\times W_{s}^{h}$ and $z\in {\tilde{W}}_{0}^{h}$:

$$\begin{array}{cc} \tilde{R}\left(s^{n+1},s^{n},w_{f}^{n+\theta };d\right) & :=R\left(s^{n+1},s^{n},w_{f}^{n+\theta };d\right)\\ & +SD_{\delta }^{n+1}\left(v_{f}^{n+\theta },w_{f}^{n+\theta },\varphi_{f}^{n+1};y,q_{f}\right)=0,\\ & R_{w_{f}}\left(w_{f}^{n+1},w_{f}^{n};z\right)=0, \end{array}$$

with the stabilization term,

$$\begin{array}{cc} & SD_{\delta }^{n+1}\left(v_{f}^{n+\theta },w_{f}^{n+\theta },\varphi_{f}^{n+1};y,q_{f}\right)\\ & =\;\int_{\Omega_{f,h}^{n+1}}\delta_{1}\;\left(\left(v_{f}^{n+\theta }\;-\;w_{f}^{n+\theta }\right)\;\cdot \;\nabla_{x}v_{f}^{n+\theta }\right)\;\cdot \;\left(\;v_{f}^{n+\theta }\cdot \nabla_{x}y\;+\;\nabla_{x}q_{f}\;\right)dx\\ & \;+\int_{\Omega_{f,h}^{n+1}}\delta_{2}\left(\nabla_{x}\cdot v_{f}^{n+\theta }\right)\cdot (\nabla_{x}\cdot y)\;dx\\ & \;+\int_{\Omega_{f,h}^{n+1}}\delta_{3}\left(\nabla_{x}\varphi_{f}^{n+1}\right)\cdot \left(v_{f}^{n+\theta }\cdot \nabla_{x}y+\nabla_{x}q_{f}\right)\;dx, \end{array}$$

and stabilization parameters,

$$\delta_{1}=4\;\rho_{f}h/v_{max},\delta_{2}=\rho_{f}h/v_{max},\delta_{3}=h/v_{max},$$

where *h* is the mesh size and *v*_*max*_ is the expected peak velocity on $\Omega_{f,h}^{0}$. Here, we replace $V^{h}$ and $M_{0}^{h}$ by ${\tilde{V}}^{h}$ and ${\tilde{M}}_{0}^{h}$, to use equal‐order interpolation for the fluid model. Then, ${\tilde{V}}_{D}^{h}$, ${\tilde{V}}_{0}^{h}$, ${\tilde{W}}_{D}^{h}$, and ${\tilde{W}}_{0}^{h}$ are given similarly to Equations (11) and (13).

### 2.5 Solver strategy

**CHeart** 1
http://cheart.co.uk

47—a multi‐physics software tool based on^5^, ^15^, ^16^, ^17^ and the matrix solver *MUMPS* 48 were used to solve the considered problem on compute nodes with 2 x Intel(R) Xeon® CPU E5‐2680 v2 (2.80 GHz) with 10 cores, 256 GB RAM and 4 × 500 GB Samsung SSD (network with 1 x QDR‐Infiniband Interconnect (40 GBit) and 1 × 1 GBit Ethernet).

Further, the Shamanskii‐Newton‐Raphson (SNR) method^49^ was employed to reduce computational cost by re‐using the Jacobian matrix (and its inverse)^5^, ^18^ as long as a sufficient decrease in the residual norm was observed. A pseudocode description of the employed algorithm is given in Algorithm 1, where *c* = 100 is used for scaling the initial residual, *γ* = 3/4 requires a sufficient residual decrease, and the general notation,

$$s^{n}=S^{n}\cdot \phi ,\;d=D\cdot \phi ,\;w_{f}^{n}=W^{n}\cdot \psi ,\;z=Z\cdot \psi ,$$

is used to define the current approximate solution and test functions (with basis functions ***ϕ*** and ***ψ***). Further, the residuals and Jacobians are denoted by

$$\begin{array}{cc} R\;(S^{n},W^{n}) & =\nabla_{D}\;R\;\left(s^{n},s^{n-1},w_{f}^{n+\theta };d\right),\\ R_{W}(W^{n}) & =\nabla_{Z}\;R\;\left(w_{f}^{n},w_{f}^{n-1};z\right),\\ J_{\beta } & =\nabla_{S}\;R\;(S^{n},W^{n}),\\ J_{W} & =\nabla_{W}\;R_{W}\;(W^{n}). \end{array}$$

Because of the iterative nature of the algorithm (Algorithm 1) and the partitioned approach, $w_{f}^{d}$ can be updated from the current solution of ***v***_*f*_ on $\Gamma_{f}^{C}$.

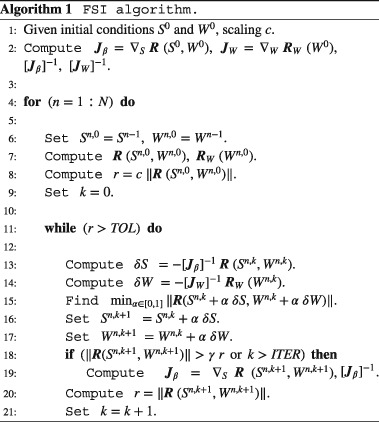

### 2.6 Error norms

Numerical results are compared with experimental data at equispaced data point positions at cross sections along the *z*‐direction with *z* ∈ {3.5, 13.5, …, 93.5}mm and 5 mm spacing along the *x*‐ and *y*‐direction, as specified in the studies of Gaddum et al^36^ and Hessenthaler et al.^37^ Further, to directly compare numerical results computed on different meshes with different mesh topology and/or geometry (Section 3.1), simulation results are sampled on a regular grid with points ***x***_*i*_, *i* = 1, …, *N*: (i) box [ − 40, 40] x [ − 40, 40] x [ − 20, 140] mm^3^ and spacing 1 mm (in each direction) for the current fluid domain Ω_*f*,*h*_; and (ii) box [ − 5.5, 5.5] x [ − 1, 1] x [0, 65] mm^3^ and spacing 0.5 mm (in each direction) for the solid domain in the reference configuration. We note that a mask is employed such that only points are considered that exist inside both fluid domains in the current configuration. For example, consider discretized domains Ω_*i*,*h*_(*t*_*p*_) and Ω_*i*,*h*_(*t*_*q*_) at times *t*_*p*_ and *t*_*q*_. Then, the mask is given as,

$$M_{i}^{pq}:=\left\{\begin{array}{ll} 1 & \;x_{i}\in \Omega_{i,h}(t_{p})\cap \Omega_{i,h}^{\prime }(t_{q}),\\ 0 & \;\text{otherwise}. \end{array}\right.$$

For comparison of results at sampled data points ***x***_*i*_, consider an approximation $w(x_{i},t_{p})\in R^{d}$ and a reference $v(x_{i},t_{q})\in R^{d}$ at times *t*_*p*_ and *t*_*q*_ with *d* ∈ {1, 3}. Then, a scalar field is defined at each point ***x***_*i*_ by computing the Euclidean distance, ∥·∥_2_, between approximation ***w*** and reference ***v***. To detect variation in the fluid and solid variables, we compute the maximum distance,

(14) $$d_{\infty }(w,v;p,q):=\max_{i=1,\text{⋯},N}M_{i}^{pq}∥w(x_{i},t_{p})-v(x_{i},t_{q}){∥}_{2},$$

and the mean distance,

(15) $$\bar{d}(w,v;p,q):=\frac{1}{N}\sum_{i=1}^{N}M_{i}^{pq}∥w(x_{i},t_{p})-v(x_{i},t_{q}){∥}_{2},$$

between an approximation and a given reference.

## 3 3D FSI BENCHMARK RESULTS AND COMPARISON WITH EXPERIMENTAL DATA

Numerical results for both phases of the 3D FSI benchmark were obtained using the inf‐sup stable (Section 2.4.2) and cG(1)cG(1) (Section 2.4.3) schemes. In this section, we present and compare the numerical results with the experimental data, while studying spatial refinement and studying how quickly steady‐state and periodic steady‐state are reached.

### 3.1 Mesh construction

**Figure 3:**
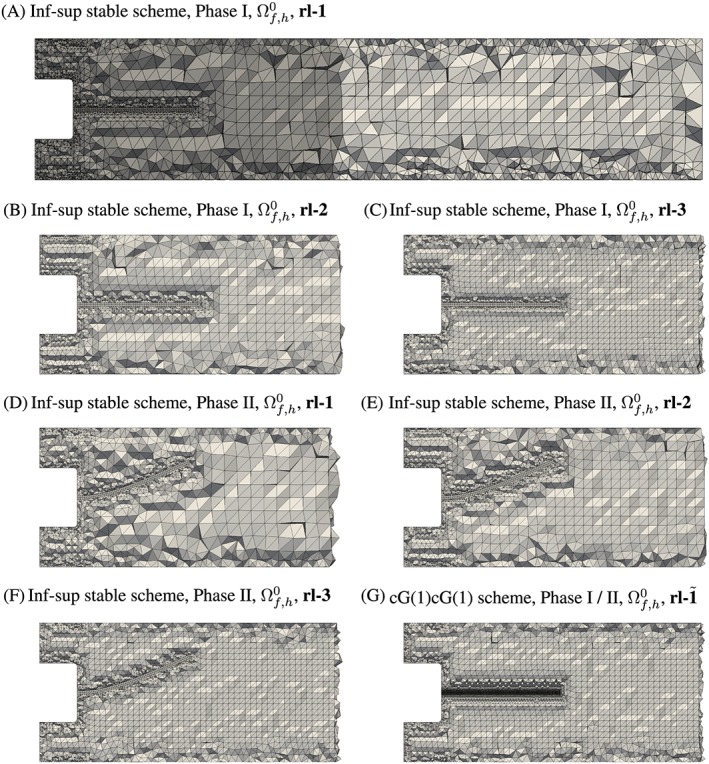
A selection of considered fluid domain discretizations shown in the reference configuration (Section 3.1), $\Omega_{f,h}^{0}$ for various refinement levels (rl), where the size of the truncated domain is indicated in dark‐gray in A, as compared to the elongated domain. Clipped domains are shown in B,‐G. Mesh details are given in Table 2

For Phase I, meshes were constructed based on undeformed geometries corresponding to a stress‐free state of the coupled FSI system and reference domains $\Omega_{f}^{0}$, $\Omega_{s}^{0}$, and $\Omega_{\lambda }^{0}$ were selected accordingly (Figure 3).

For Phase II, the Phase I meshes were deformed based on the coupled FSI system being exposed to gravity loading of 70*%* strength. Then, fluid and interface domains were remeshed, to counteract deteriorating mesh quality due to large mesh deformations, and the fluid and reference domains redefined to be in this deformed state (Figure 3). Finally, the coupled FSI system was subjected to 100*%* gravity loading to obtain a deformed state that matches the state during the calibration step of Phase II of the FSI experiments.^37^

**Figure 4:**
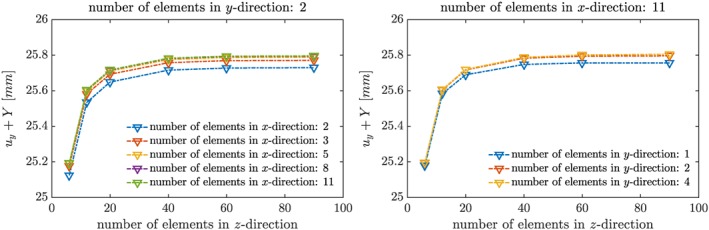
The graph depicts the position of the center of the solid tip, *u*_*y*_ + *Y*, under static gravity loading depending on mesh resolution, where the number of elements in one coordinate direction is kept fixed and varied in the other coordinate directions

In preliminary numerical experiments, the static deflection of the solid in its hydrostatic equilibrium (with material parameters for Phase II; Table 1) was studied regarding mesh resolution. Buoyancy forces in a surrounding resting fluid were mimicked by applying the net gravity load, specifically (*ρ*_*s*_ − *ρ*_*f*_)/*ρ*_*s*_ ***g***. Equation (6) was used for the solid model, but the inertial term was neglected (because predictions of a static and transient model are expected to tend to the same asymptotic limit subject to static loading conditions). As expected, because of the high‐aspect ratio of the solid side lengths, the observed maximum deflection of the tip of the solid is predominantly influenced by the mesh size in *z*‐direction (Figure 4). A similar result is anticipated for dynamic loading cases, such that a fine mesh resolution of 11 × 2 × 65 hexahedral elements seems a good choice for both Phases I and II.

For the model given in Section 2.4.2, the fluid domain boundary Γ_*f*_ was discretized using a fine triangular surface mesh. On the coupling boundary $\Gamma_{f}^{C}$, the triangular mesh was constructed, such that a quadrilateral surface element on $\Gamma_{s}^{C}$ would have two triangular surface elements as counterparts on $\Gamma_{f}^{C}$ with matching nodes. Aim of selecting a fixed but fine mesh resolution on Γ_*f*_ was to avoid dominant boundary effects while studying spatial refinement in the interior of the domain, where three different mesh refinement levels were considered (referred to as rl‐1, rl‐2, rl‐3), see Figure 3 and Table 2. On the other hand, only one refinement level rl‐ $\tilde{1}$ was considered for the model given in Section 2.4.3 (Figure 3G), as we expect similar behavior with respect to spatial refinement. Here, the fluid coupling boundary $\Gamma_{f}^{C}$ was discretized using triangle elements, such that a quadrilateral surface element on $\Gamma_{s}^{C}$ would have two triangular surface elements as counterparts on $\Gamma_{f}^{C}$.

**Table 2:** Mesh details for flow and Lagrange multiplier domains, Ω_*f*_ and Ω_*λ*_

		**Refinement**	**Number of elements**		**Number of nodes**		**Avrg. runtime**	
Phase	Scheme	**level**	$N_{f}^{e}$ // $N_{\lambda }^{e}$	*v*_*f*_	*φ*_*f*_	*λ*	**per time step**	Number of cores
I	inf‐sup st.	rl‐1	127 774 // 3424	195 635	27 914	6875	13.89 s	16
	inf‐sup st.	rl‐2	158 236 // 3424	236 137	32 934	6875	22.10 s	16
	inf‐sup st.	rl‐3	333 048 // 3424	469 487	62 203	6875	38.72 s	16
	cG(1)cG(1)	rl‐ $\tilde{1}$	673 066 // 54 784	133 663	133 663	27 445	21.01 s	16
	inf‐sup st.	rl‐1‐trunc	135 352 // 3424	207 431	29 615	6875	10.39 s	16
	inf‐sup st.	rl‐2‐trunc	152 126 // 3424	229 725	32 375	6875	13.04 s	16
II	inf‐sup st.	rl‐1	131 263 // 3424	200 276	28 490	6875	12.60 s	16
	inf‐sup st.	rl‐2	166 205 // 3424	246 812	34 287	6875	14.86 s	16
	inf‐sup st.	rl‐3	351 221 // 3424	493 664	65 205	6875	38.72 s	16
	cG(1)cG(1)	rl‐ $\tilde{1}$	673 066 // 54 784	133 663	133 663	27 445	50.53 s	32
	inf‐sup st.	rl‐1‐trunc	136 955 // 3424	209 562	29 879	6875	13.16 s	16
	inf‐sup st.	rl‐2‐trunc	160 539 // 3424	241 002	33 807	6875	14.61 s	16

The solid domain was discretized using 1430 elements with 15 065 nodes for ***u***_*s*_ and 2376 nodes for *φ*_*s*_.

We note that the outlet of the computational domain was extended by 250 mm to regularize flow downstream. To study the necessity of such an elongation, a truncated domain (Figure 3A) in combination with the model given in Section 2.4.2 was also considered with two refinement levels (referred to as rl‐1‐trunc and rl‐2‐trunc) for Phases I and II, respectively.

The triangular surface mesh on the fluid domain's coupling boundary was extracted from the mesh discretizing $\Omega_{f}^{0}$ to define the mesh on Ω_*λ*_.

### 3.2 Phase I experiment

Initially, the FSI system is at rest and subject to no body forces (no flow, no displacement). Then, parabolic inflow is prescribed at both inlets as given in Equation (1) (Figure 2A). Simultaneously, gravitational forces are increased according to (24*t* ^3^ − 8*t* ^2^) · ***g*** over 0.5 s and kept constant at 1 · ***g*** for *t* ⩽ 0.5 s. Because a steady‐state is expected,^37^ an increasing sequence of time step sizes was employed; eg, we simulated 2 s with Δ_*t*_ = 1 ms, 60 s with Δ*t* *′* = 10 ms, and 138 s with Δ*t*″ = 100 ms (such that the total simulated time was *T* = 200 s).

Because of increased inflow and gravitational forces, the silicone filament moves into the way of the incoming flow jet. The impact causes strong disturbance in the flow field, which is then advected toward the outlet causing reflow regions at the outflow boundary. Here, backflow divergence is avoided effectively by employing outflow stabilization (Equation (9)).

**Figure 5:**
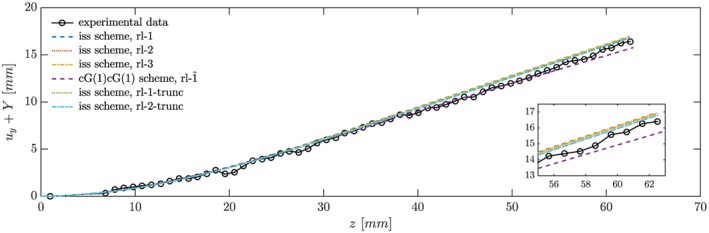
Phase I: Final position of the centerline of the silicone filament is predicted well by the inf‐sup stable (Section 2.4.2) and cG(1)cG(1) (Section 2.4.3) scheme

**Figure 6:**
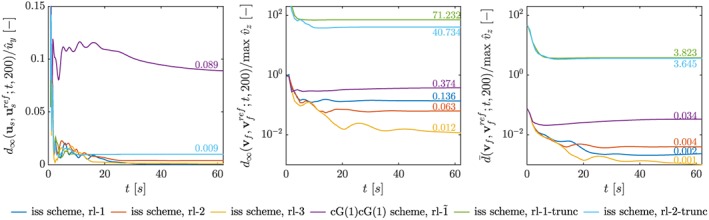
Phase I: The graph depicts the maximum and mean Euclidean distance, *d*_*∞*_ and $\bar{d}$ (Equations (14) and (15)), where ${ũ}_{y}\approx 16.41$ mm is the maximum deflection observed in the experiment and ${\tilde{v}}_{z}=630$ mm/s the recorded maximum inflow.^37^ Here, we selected $\left(u_{s}^{\text{ref}},v_{f}^{\text{ref}}\right)$ as the final state obtained with the inf‐sup stable scheme, rl‐3

After a short transition phase, the silicone filament reaches a steady position and flow stabilizes. Figure 5 illustrates that the final position of the silicone filament along its centerline is predicted consistently by the inf‐sup stable scheme with a slightly overestimated deflection. On the other hand, the cG(1)cG(1) scheme slightly underestimates the deflection of the silicone filament near the tip. However, agreement with the experimental data is very good considering finite voxel sizes in MRI.^37^ To investigate how quickly the silicone filament reaches its steady‐state position (where the position predicted by the inf‐sup stable scheme with fluid domain refinement level 3 is assumed as the steady‐state position), we employ Equation (14) to quantify the maximum Euclidean distance to the steady‐state position. Figure 6 shows that the solid reaches its final position quickly irrespective of the fluid domain mesh resolution or employed scheme, such that the fluid domain deformation is negligible after the initial transition phase.

**Figure 7:**
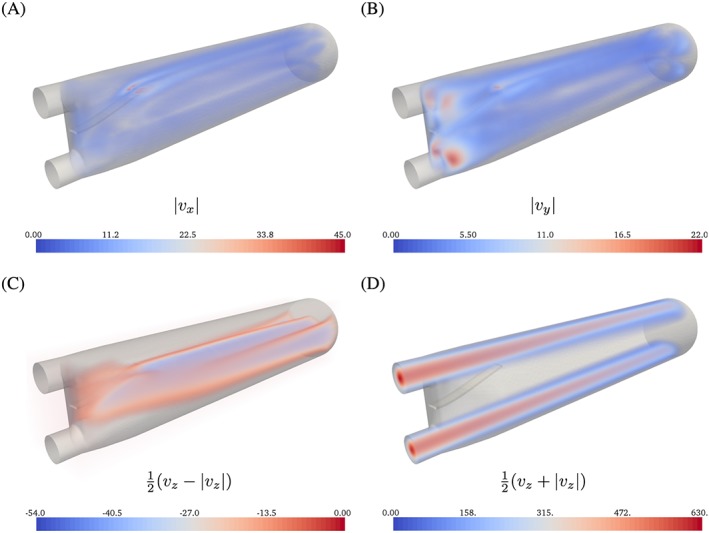
Phase I: Flow in the *v*_*x*_ and *v*_*y*_ components A, and B, is significantly smaller than in the *v*_*z*_ component because of the incoming flow C, and D. Here, opacity for volume rendered flow components ranges from 0 to 1 in A, B, and D, and from 1 to 0 in C

**Figure 8:**
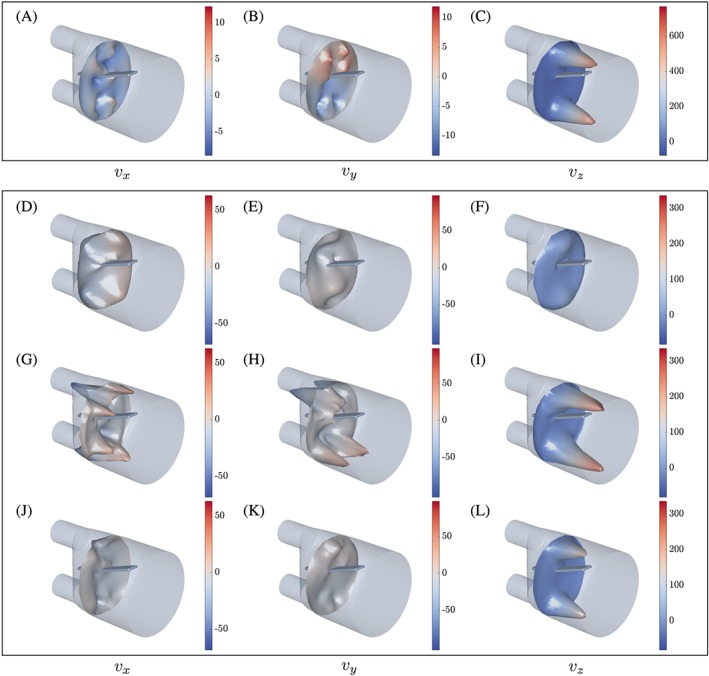
Predicted flow velocity components at *z* = 30 mm, obtained using the inf‐sup stable scheme, rl‐3. A‐C, Phase I at *t* = 200 s. Phase II at D‐F, *t* = 0.72 s; G‐I, *t* = 1.20 s; J‐L, *t* = 2.64 s. Flow patterns exhibit significant similarities with those observed in the experiment (see the study of Hessenthaler et al^37^)

Once flow has stabilized and fluctuations become smaller, flow in the *v*_*x*_ and *v*_*y*_ components is less pronounced (Figure 7) than during the transition phase. Flow mainly occurs in the *v*_*z*_ component, and the presence of the silicone filament does not seem to cause significant disturbance or deflection of the flow jets entering from the inlets. In fact, the tip of the silicone filament is positioned just below the upper flow jet. Between the two flow jets, a large recirculation zone is observed, where approximately one third of the outflow boundary is covered by fluid flowing back into the computational domain, see Figure 7C,D. Investigating differences in the flow predictions obtained for various fluid domain refinement levels using both schemes, it becomes clear that an early truncation of the domain can change local flow in the considered region significantly, see Figure 6. Further, flow predictions computed for the elongated fluid domain are consistent for all meshes indicating that rl‐1 provides a sufficient refinement. Similarly to the solid deflection, a steady‐state flow field is obtained quickly (Figure 6), such that the simulated time can be reduced drastically without corrupting the approximation accuracy of the predicted steady‐state. Finally, comparing flow predictions and experimental results at plane *z*≈ 30 mm shows good qualitative agreement for all velocity components, see Figure 8A‐C.

### 3.3 Phase II experiment

Initially, the FSI system is at rest with the initial configuration corresponding to the hydrostatic equilibrium (that is, solid deformation due to gravitational forces). Parabolic inflow is prescribed at both inlets with peak values fit to experimental data (Figure 2B). A total of *K* = 10 cycles of the periodic inflow pulse seen in Figure 2B were simulated with time step size 1 ms. In the following, we refer to each cycle individually by using the notation $t_{k}^{n}\in [0,6]$ s (with *k* = 1,2,…,10 and *n*  =  0,1, … ,6000) to simplify comparison of corresponding time steps of two different cycles, eg, cycles k and K with $t_{k}^{n}$ and $t_{K}^{n}$.

**Figure 9:**
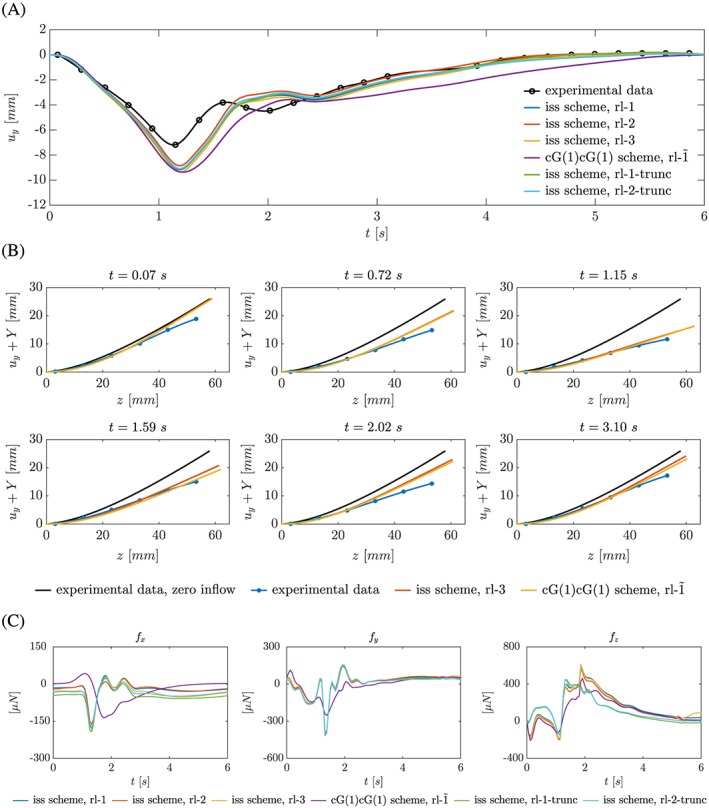
Phase II: A, Graph depicts the recorded relative displacement and predicted relative displacement (cycle 10) of the silicone filament at *x* = 0 mm, *z* ≈ 53 mm for all refinement levels and both schemes. The experimental data were approximated using spline interpolation. B, Graph shows snapshots of recorded position and predicted position (cycle 10, inf‐sup stable scheme, rl‐3) of the centerline of the solid at *x* = 0 mm (graph from the study of Hessenthaler et al^37^ modified). C, Forces ***f*** exerted onto the solid during cycle 10 computed from the Lagrange multiplier variable (Equation (16))

Starting from its initial position at the beginning of each cycle (referred to as *resting position*), the silicone filament is pushed downwards because of the impacting flow jet entering from the upper inlet (the time‐dependent position of the solid's centerline is given in Figure 9). Once flow decelerates because of decreased inflow, gravitational forces become more dominant, and the silicone filament moves back upwards performing a swing before reaching its resting position. The silicone filament follows the same repeatable deflection pattern as observed in the experiment; however, its resting position coincides with the deflection under zero inflow conditions, which was not found in the experiment. The relative displacement of the silicone filament (Figure 9A) is predicted well. Even though the maximum relative displacement at *t* ≈ 1.15 s is overestimated by approximately 1.8 mm (on average), the instant when the maximum relative displacement is reached is predicted precisely and the swing at *t* ≈ 1.59 s governed (however, a short delay is observed). While the deflection pattern is consistently predicted by the inf‐sup stable scheme on all refinement levels (irrespective of fluid domain length), the cG(1)cG(1) scheme yields a slightly different relative displacement pattern with a lagging solid motion, thus the silicone filament reaching its resting position later (Figure 9A). Further, the swing is less pronounced. We note that the numerical results in Figure 9B are Lagrangian positions. On the other hand, the position of the solid under flow conditions was obtained from intersecting the solid's centerline with MRI image planes at *Z*_*s*_ ≈ 3 mm + *s*·10 mm, *s* =  0, …, 5. Thus, the position of the solid is not available for its entire length. Similarly, the position of the solid under zero inflow conditions was extracted from an MRI image plane at *x* ≈ 0 mm.

**Figure 10:**
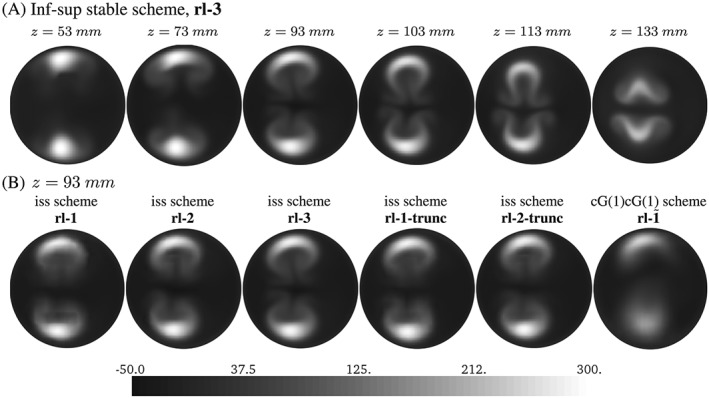
Phase II: Velocity *v*_*z*_ at cross sections at *t* = 2.04 seconds. As was observed in the experiment,^37^ a double Ω‐shaped flow pattern develops from the two flow jets entering the flow domain, see Figure 11A

**Figure 11:**
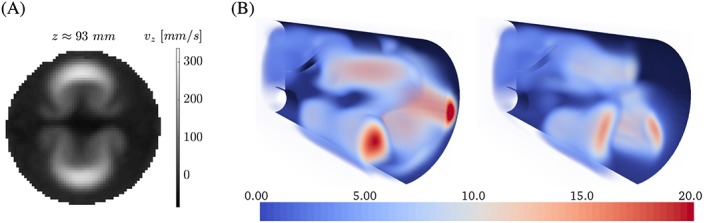
A, MRI measurements^37^: Velocity *v*_*z*_ at *t* ≈ 2.02 s. B, Phase II: Velocity *v*_*y*_ at *t* = 3.96 s (volume rendered with opacity ranging from 0 to 1) obtained with the inf‐sup stable scheme and the elongated (left; rl‐3) and the truncated computational domain (right; rl‐1). Here, both domains are truncated at *z* = 160 mm to simplify comparison

Flow patterns show strong similarities with those observed in the experiment. For example, a double Ω‐shape is observed at flow planes that drifts in negative *x*‐direction along the *v*_*z*_‐direction (Figure 10A) because of the non‐zero *v*_*x*_‐component at the inflow boundary. Further, the flow dynamics and local flow phenomena are governed well and compare well with the experimental data 2
http://cheart.co.uk/other-projects/fsi-benchmark/
(compare Figure 8D‐L and the study of Hessenthaler et al^37^). We note that minor differences in the local flow velocities are observed if the flow domain is truncated as exemplified for the *v*_*y*_‐component in Figure 11B. For example, finer resolution yields a more symmetric prediction of the flow field, and stronger flow in the upward direction is observed at the truncation and near the tip of the silicone filament. However, it does not affect the position of the silicone filament (Figure 9A).

Forces exerted onto the solid were computed by integrating the Lagrange multiplier variable (which represents the traction on the coupling boundary Ω_*λ*_),

(16) $$f={\left[\;f_{x},f_{y},f_{z}\right]}^{T}=\int_{\Omega_{\lambda }}(\lambda \cdot n_{\lambda })\cdot n_{\lambda }+(\lambda \cdot t_{\lambda })\cdot t_{\lambda }+(\lambda \cdot s_{\lambda })\cdot s_{\lambda }\;dx,$$

where ***n***_*λ*_, ***t***_*λ*_, and ***s***_*λ*_ are linearly independent normal and tangent unit vectors on Ω_*λ*_. As Figure 9C illustrates, the *f*_*y*_ component is negative for downward motion of the silicone filament and positive when it starts moving upwards after the largest relative displacement. For the *f*_*x*_ and *f*_*y*_ components, the truncation of the domain does not induce significant differences; however, the maximum force in *f*_*z*_‐direction during cycle 10 is observed earlier. Forces predicted by the cG(1)cG(1) scheme are significantly different; however, match with observations of relative solid motion (for example, the smaller *f*_*y*_ force after the largest relative displacement is in good agreement with the silicone filament moving toward its resting position more slowly).

**Figure 12:**
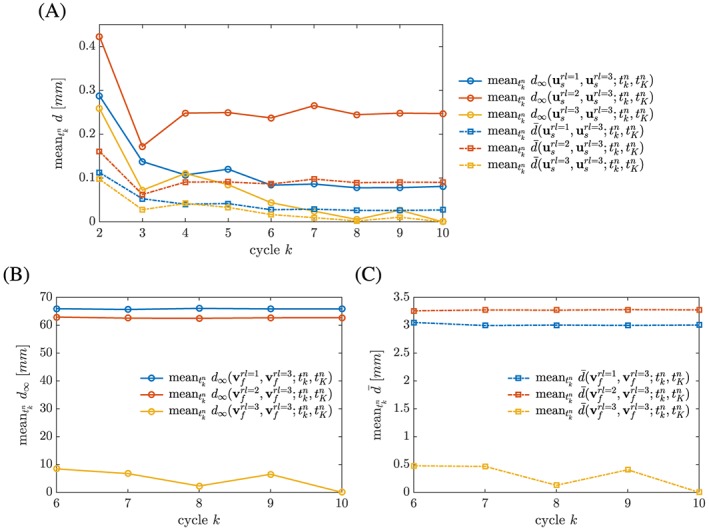
Phase II: The graph quantifies how quickly a periodic steady‐state is obtained for the displacement and flow field, ***u***_*s*_ and ***v***_*f*_, depending on the refinement level in the fluid domain. Displacement and velocity, $u_{s}^{rl}$ and $v_{f}^{rl}$, at given fluid domain refinement level for an intermediate cycle *k* is compared with displacement and velocity, $u_{s}^{rl\mathrm{=3}}$ and $v_{f}^{rl=3}$, at rl‐3 for final cycle *K* = 10 using error norms given in Equations (14) and (15) and computing the mean for given cycle $k\left(\text{note},t_{k}^{n}=t_{K}^{n}\right)$

To investigate how quickly the FSI system reaches a periodic steady‐state, we employ Equations (14) and (15) to detect fluctuations in the solid motion and flow (assuming cycle 10 with the inf‐sup stable scheme and rl‐3 as the periodic steady‐state). Here, we consider the inf‐sup stable scheme and refinement levels 1‐3. As Figure 12A illustrates, the solid deformation reaches a periodic steady‐state after a small number of cycles on all refinement levels, and the same holds for the flow field, see Figure 12B,C. Although Figure 12B indicates that a different periodic steady‐state flow field was found on refinement levels 1 and 2, the quite large error is related to insufficient fluid domain refinement in regions of large velocity gradients (for example, note the more diffused appearance of the double Ω‐shape in Figure 10B) such that the error norm given in Equation (15) is a much more suitable measure of error in the predicted flow field (Figure 12C).

An increased average runtime for Phase II was observed for the cG(1)cG(1) scheme (Table 2), which arises from more Newton iterations per solve step. This stems from a general delay in rebuilding the Jacobian matrix (Algorithm 1). However, we note that the SNR solver parameters were tailored for the inf‐sup stable approach and subsequently used for both methods. Runtime would likely improve significantly for the cG(1)cG(1) approach with adaption of SNR solver.

## 4 DISCUSSION AND CONCLUSION

An inf‐sup stable FSI scheme has been validated and compared with, and a stabilized cG(1)cG(1) scheme (presented in Sections 2.4.2 and 2.4.3) has been validated using benchmark data obtained from a recently developed 3D FSI experiment.^37^

For the Phase I experiment, numerical results for predicted solid displacement and flow velocity were found to agree exceptionally well with available experimental data with solid deflection slightly overestimated/underestimated by the inf‐sup stable / cG(1)cG(1) scheme. On the other hand, numerical results for the Phase II experiment yield a good prediction of flow patterns (eg, Figure 10A), but it was found that a fine mesh resolution is required to sufficiently resolve flow regions with large velocity gradients (Figure 10B).

Overall, the inf‐sup stable scheme performed better in the considered cases regarding prediction of relative solid displacement or shape of flow patterns. Although, employed mesh resolution for the cG(1)cG(1) can be considered coarse.

It is recommended to simulate Phase I for about 30 seconds and Phase II for at least 5 cycles to reach an approximate (periodic) steady‐state with sufficient accuracy (with respect to initial / boundary conditions and error norms employed in this paper, eg, no significant fluctuations in solid motion and flow are observed; Figures 6 and 12). Results show that an elongation of the computational domain by 250 mm yields significantly better accuracy than an elongation by 50 mm. Further, mesh resolution has to be tuned depending on the phase of the benchmark because dynamic effects need to be governed in Phase II, thus requiring a finer fluid domain discretization as compared to Phase I. However, computational cost depends on the selected spatial mesh refinement of the fluid domain, as can be appreciated in Table 2. We note that the increased average runtime for Phase II in the case of the cG(1)cG(1) scheme stems from an observed increase in the number of Newton iterations. For the cG(1)cG(1) scheme, parameters to trigger rebuilding the Jacobian (see Algorithm 1) were chosen based on the inf‐sup stable scheme, however, delay the rebuild step in the transient test case and have thus to be tuned for better performance. From numerical experiments presented in this paper, we conclude that solid motion is not significantly altered by fluid mesh resolution; however, solid motion is influenced by the selected scheme.

Future investigations on the considered 3D FSI benchmark will include, for example, studying temporal convergence, sensitivity to the selected solid material law, spatial mesh refinement for the cG(1)cG(1) scheme, a second‐order time‐integration scheme, and sensitivity to outflow stabilization (preliminary results with *β* = 0.02 indicate no dependency on the outflow stabilization; however, final observations are likely to depend on the selected fluid domain length). Further, computational cost can potentially be decreased by optimizing the length of the computational domain, using a coarser solid mesh resolution and making use of the symmetry in the Phase I setup.
